# Retraction Note: Long noncoding RNA MIR31HG inhibits hepatocellular carcinoma proliferation and metastasis by sponging microRNA-575 to modulate ST7L expression

**DOI:** 10.1186/s13046-025-03340-8

**Published:** 2025-02-25

**Authors:** Shaoying Yan, Zhenrong Tang, Ke Chen, Yuyang Liu, Gangfeng Yu, Qiuxu Chen, Hao Dang, Fengjiao Chen, Jiaji Ling, Liying Zhu, Ailong Huang, Hua Tang

**Affiliations:** 1 Key Laboratory of Molecular Biology for Infectious Diseases (Ministry of Education), Institute for Viral Hepatitis, Department of Infectious Diseases, The Second Affiliated Hospital, Chongqing Medical University, 1 Yi Xue Yuan Road, Chongqing, 400016 China; 2 Department of Endocrine and Breast Surgery, The First Affiliated Hospital of Chongqing Medical University, Chongqing, China; 3 Collaborative Innovation Center for Diagnosis and Treatment of Infectious Diseases, Zhejiang University, Hangzhou, China

**Retraction Note** **:** **J Exp Clin Cancer Res 37, 214 (2018)**

**https://doi.org/10.1186/s13046-018-0853-9**

The Editor-in-Chief has retracted this article. After publication, concerns were raised regarding highly similar images in the figures, specifically:

- Fig. 4g SMMC7721 pre-NC image appears to overlap with Fig. 6d SMMC7721 sh-MIR31HG+anti-miR-575;
- Fig. 4h SMMC7721 pre-NC image appears to overlap with Fig. 6e SMMC7721 pcDNA3.1-MIR31HG+pre-miR-575.

The authors checked their data and found that in Fig. 2c, SMMC7721 pcDNA3.1-MIR31HG and sh-MIR31HG 0h images were the same, and Fig. 2c HepG2 pcDNA3.1-MIR31HG 36h and Fig. 4f HepG2 anti-NC 0h images originated from the same sample. Further checks by the Publisher have also identified a potential error in Fig. 6e, where HepG2 sh-MIR31HG+anti-miR-575 and pcDNA3.1+pre-NC images appear to originate from the same sample.

The authors requested a Correction and provided the original image files and cell line authentication documents. However, the STR profiling results of the cell lines used in the *in vitro* and *in vivo* experiments this study revealed that the SMMC7721 and L-02 cell lines were contaminated with HeLa cervical cancer cells, making them an unsuitable model for liver cancer. This partially undermines the main conclusions of the article, which are specific to hepatocellular carcinoma.

The Editor-in-Chief therefore no longer has confidence in the presented data and conclusions of this artilcle.

Shaoying Yan and Hua Tang disagree with this retraction. None of the other authors have responded to any correspondence from the Publisher about this retraction.

